# Revised North Star Ambulatory Assessment for Young Boys with Duchenne Muscular Dystrophy

**DOI:** 10.1371/journal.pone.0160195

**Published:** 2016-08-05

**Authors:** Eugenio Mercuri, Giorgia Coratti, Sonia Messina, Valeria Ricotti, Giovanni Baranello, Adele D’Amico, Maria Carmela Pera, Emilio Albamonte, Serena Sivo, Elena Stacy Mazzone, Maria Teresa Arnoldi, Lavinia Fanelli, Roberto De Sanctis, Domenico M Romeo, Gian Luca Vita, Roberta Battini, Enrico Bertini, Francesco Muntoni, Marika Pane

**Affiliations:** 1 Department of Paediatric Neurology, Catholic University, and Nemo Roma center for neuromuscular disorders, Rome, Italy; 2 Department of Neurosciences and Nemo and Clinical Center, Psychiatry and Anaesthesiology, University of Messina, Messina, Italy; 3 Dubowitz Neuromuscular Centre, Institute of Child Health, University College, London, United Kingdom; 4 Developmental Neurology Unit, Istituto Neurologico “Besta”, Milan, Italy; 5 Unit of Neuromuscular and Neurodegenerative Diseases, Department of Neurosciences, Bambino Gesù Children's Hospital, Rome, Italy; 6 Department of Developmental Neuroscience, Stella Maris Institute, Pisa, Italy; IRCCS-Policlinico San Donato, ITALY

## Abstract

The advent of therapeutic approaches for Duchenne muscular dystrophy (DMD) has highlighted the need to identify reliable outcome measures for young boys with DMD. The aim of this study was to develop a revised version of the North Star Ambulatory Assessment (NSAA) suitable for boys between the age of 3 and 5 years by identifying age appropriate items and revising the scoring system accordingly. Using the scale in 171 controls between the age of 2.9 and 4.8 years, we identified items that were appropriate at different age points. An item was defined as age appropriate if it was completed, achieving a full score, by at least 85% of the typically developing boys at that age. At 3 years (±3months) there were only 8 items that were age appropriate, at 3 years and 6 months there were 13 items while by the age of 4 years all 17 items were appropriate. A revised version of the scale was developed with items ordered according to the age when they could be reliably performed. The application of the revised version of the scale to data collected in young DMD boys showed that very few of the DMD boys were able to complete with a full score all the age appropriate items. In conclusion, our study suggests that a revised version of the NSAA can be used in boys from the age of 3 years to obtain information on how young DMD boys acquire new abilities and how this correlates with their peers.

## Introduction

The recent development of therapeutic approaches for Duchenne Muscular Dystrophy (DMD) has highlighted the need to identify clinical outcome measures for clinical trials [1–3]. So far most of the trials have focused on boys above the age of 5 years. With the first completed clinical trials showing promising results however there is increasing pressure from advocacy groups, clinicians, industries and regulatory authorities, to start possible treatments when the disease is still in the early phase, before muscle tissue becomes progressively replaced.

Little however has been reported on how to assess young DMD boys before the age of 5 years, and more generally on early neurodevelopmental and motor aspects in preschool DMD boys [4–5]. This is mainly due to the fact that although DMD children already show some signs of developmental delay and inability to develop new motor abilities by the age of 2 years, age at diagnosis is still on average above the age of four years.

Only recently, using neurodevelopmental scales (Bayley scale in US and Griffiths scales in Italy/UK), two studies have provided information on the profile of developmental difficulties in young DMD boys. These studies showed similar findings. Gross motor and speech delay were often the presenting signs. With increasing age, DMD boys acquire new skills and improve the ability to perform some motor activities while other activities, such as running fast or hopping are not commonly achieved in DMD boys not treated with steroids. Even if boys acquire new skills, however, when compared to their peers, repeated measures analysis reveal that gross motor scores, decline over time as they do not approach the gross motor skills expected for typically developing boys of the same age. Scales like the Bayley however can only be used up to the age of 36 months and there is therefore a gap between this age and the age of 5 years, when validated measures and natural history data are available [4–5].

There have been suggestions that the North Star Ambulatory Assessment (NSAA), a functional scale specifically developed for assessing motor function in DMD, and validated in ambulant DMD children older than 5 years, may be used in younger children [6].

In a recent study, we used the NSAA in typically developing preschool boys at different ages from the age of 3 in order to assess which are the activities that are not consistently achieved at different age points (3 years, 3.5 years, 4 years etc). The results showed that the scale, in its original version including 17 items, could be used from the age of 4 years but that before the age of 4, many items, such as standing on one leg or hopping, were not suitable as these assessed activities that were not always achieved by typically developing boys at that age [6].

The aim of this study was to develop a revised version of the NSAA suitable for boys between the age of 3 and 5 years. More specifically we wished to:

i) expand the previous work on typically developing boys in order to better define the items that are developmentally appropriate before the age of 5 years; ii) develop a revised version that, at each age, only includes age appropriate items and revise the scoring system accordingly; iii) apply this scoring system to available NSAA data obtained in young DMD boys.

## Subjects and Methods

The study is a prospective multicentric study involving four tertiary neuromuscular centers in Italy and one in United Kingdom.

The study was approved by the Ethics committee in each center (Catholic University and Ospedale Bambino Gesù, Rome; University of Messina, Messina; Stella Maris Institute, Pisa; Neurological Institute C. Besta, Dubowitz Neuromuscular Centre, London). As the assessments were already part of the clinical routine in all centers, consent to anonymously record the data in a database was obtained by the parents for the boys under age. For the typically developing boys, parents signed an informed consent to participate to the study. Data was collected for both cohorts between January 2013 and June 2015.

### Control group

171 controls between the age of 3 and 5 years were examined in their schools by six examiners from 3 centers (Rome, Milan, Pisa). Parents and teachers were asked if the child was born prematurely or if there was any previous concern about their development leading to a referral to a pediatrician or a neurologist. When this occurred, children were assessed but their data were not included in the analysis. Their age ranged from 2 years, 9 months to 4 years and 8 months.

### DMD

173 assessments from 75 DMD boys younger than five years and with a genetically proven DMD diagnosis were included. As diagnosis was performed often around the age of 4 years, not all boys had serial longitudinal assessments. In order to make sure that the abilities achieved were not the result of pharmacological intervention boys on steroids were excluded.

### NSAA

The scale is an ordinal scale consisting of 17 items, ranging from standing (item 1) to running (item 17). It includes several items assessing abilities that are necessary to remain functionally ambulant, items assessing abilities, such as head raise and standing on heels that can be partly present in the early stages of the disease and a number of activities such as hopping, or running that are generally never fully achieved in untreated DMD boys but that have been found in those treated with daily steroids.

Each item can be scored on a 3 point scale using simple criteria: 2 –Normal achieves goal without any assistance; 1 –Modified method but achieves goal independent of physical assistance from another person; 0 –Unable to achieve independently.

A total score can be achieved by summing the scores for all the individual items. The score can range from 0, if all the activities are failed, to 34, if all the activities are achieved.

Details of the training for the physiotherapists involved in the study and of the interobserver reliability for NSAA among the centers have already been reported [7–11].

### Defining age specific items

Data on the NSAA in controls at the age younger than 5 years were already available from our recent study, but we increased the number in each subgroup, using the same assessors, the same testing procedures and the same settings used in the previous study [6]. The only difference in recruitment is that we expanded the lower age level as the youngest group included boys of age 3 years ± 3 months and excluded those at age 5 years as by this age many boys had started steroid treatment. The data was analyzed subdividing the cohort into boys assessed at 3, 3.5, 4 and 4.5 years (± 3 months for each subgroup).

A frequency distribution was calculated for each item at each age point, using a cut-off point of 85%. An item was defined as age appropriate if it was completed, achieving a full score, by at least 85% of the typically developing boys at that age. The cut-off point of 85% was arbitrarily chosen among those commonly used as in many tests 15th centile is often used to define values outside the normal range.

At each age point, only the items considered age appropriate were selected and contributed to the maximum total score for that age group.

In order to be sure that our findings in the controls were not discordant with reported developmental milestones, the results obtained in the control group were compared to a number of developmental scales, including the Griffiths mental scales, the Denver developmental scales, Alberta Infant Motor scales [12–14] for which age related reference data were available.

### Application of the new scoring system to the DMD data

Cross sectional data were available for 173 assessments from 75 boys who had at least one assessment before the age of 5 years. At each age point, only the items that had been identified as age appropriate were selected. The total score achieved was then expressed as % predicted in relation to the maximum total score achievable at that age.

### Statistical analysis

As the DMD and typically developing boys were matched for age (p>0.05) and sex, the comparison of the results for each item was assessed by using the Wilcoxon matched pairs signed-ranks test.

Level of significance was set at p<0.05.

## Results

### Control group

At age 3, full scores were consistently achieved by 85% of the controls only in 8 items (1,2,3,6,7,10,14,17) with a maximum age appropriate total score of 16.

At age 3.5 years full scores were achieved by 85% of the controls in 5 additional items (4,5,8,9,13). The number of age appropriate items increased to 13, with a maximum age appropriate total score of 26.

By age 4 years, full scores were achieved in 85% of the controls in all 17 items (including 11,12,15,16) with a maximum age appropriate total score of 34, both at 4 and at 4.5 years.

Table 1 shows details of the frequency distribution for each item in the control group.

**Table 1:** Frequency distribution of controls reaching a full score on individual NSAA items at different age points. The shaded cells indicate the activities that were achieved by at least 85% of the boys at that age point and were therefore considered as age appropriate.

	North Star	CONTROLS 3 years (n:35)	CONTROLS 3.5 years(n:51)	CONTROLS 4 years(n:33)	CONTROLS 4.5 years(n:52)
*1*	Stand	Full score: 100%	Full score: 100%	Full score: 100%	Full score: 100%
		Mean: 2	Mean: 2	Mean: 2	Mean: 2
		St.Dv: 0	St.Dv: 0	St.Dv: 0	St.Dv: 0
*2*	Walk(10m)	Full score: 100%	Full score: 100%	Full score: 100%	Full score: 100%
		Mean: 2	Mean: 2	Mean: 2	Mean: 2
		St.Dv: 0	St.Dv: 0	St.Dv: 0	St.Dv: 0
*3*	Sit to stand from chair	Full score: 100%	Full score: 100%	Full score: 100%	Full score: 100%
		Mean: 2	Mean: 2	Mean: 2	Mean: 2
		St.Dv: 0	St.Dv: 0	St.Dv: 0	St.Dv: 0
*6*	Climb step—R	Full score: 86%	Full score: 98%	Full score: 97%	Full score: 98%
		Mean: 1.857	Mean: 1.980	Mean: 1.969	Mean: 1.980
		St.Dv: 0.355	St.Dv: 0.140	St.Dv: 0.174	St.Dv: 0.138
*7*	Climb step—L	Full score: 86%	Full score: 96%	Full score: 100%	Full score: 100%
		Mean: 1.828	Mean: 1.960	Mean: 2	Mean: 2
		St.Dv: 0.452	St.Dv: 0.196	St.Dv: 0	St.Dv: 0
*10*	Gets to sitting	Full score: 97%	Full score: 100%	Full score: 97%	Full score: 100%
		Mean: 1.971	Mean: 2	Mean: 1.969	Mean: 2
		St.Dv: 0.169	St.Dv: 0	St.Dv: 0.174	St.Dv: 0
*14*	Jump	Full score: 94%	Full score 96%	Full score: 100%	Full score: 100%
		Mean: 1.914	Mean: 1.960	Mean: 2	Mean: 2
		St.Dv: 0.373	St.Dv: 0.196	St.Dv: 0	St.Dv: 0
*17*	Run	Full score: 86%	Full score: 88%	Full score: 97%	Full score: 100%
		Mean: 1.882	Mean: 1.882	Mean: 1.969	Mean: 2
		St.Dv: 0.327	St.Dv: 0.325	St.Dv: 0.174	St.Dv: 0
*4*	Stand on one leg—R	Full score: 66%	Full score: 88%	Full score: 97%	Full score: 100%
		Mean: 1.657	Mean: 1.882	Mean: 1.969	Mean: 2
		St.Dv: 0.481	St.Dv: 0.325	St.Dv: 0.174	St.Dv: 0
*5*	Stand on one leg—L	Full score: 69%	Full score: 86%	Full score: 97%	Full score: 100%
		Mean: 1.685	Mean: 1.862	Mean: 1.969	Mean: 2
		St.Dv: 0.471	St.Dv: 0.347	St.Dv: 0.174	St.Dv: 0
*8*	Descend step—R	Full score: 74%	Full score 94%	Full score: 100%	Full score 98%
		Mean: 1.714	Mean: 1.941	Mean: 2	Mean: 1.980
		St.Dv: 0.518	St.Dv: 0.237	St.Dv: 0	St.Dv: 0.138
*9*	Descend step—L	Full score: 63%	Full score: 88%	Full score: 94%	Full score: 100%
		Mean: 1.514	Mean: 1.862	Mean: 1.909	Mean: 2
		St.Dv: 0.701	St.Dv: 0.401	St.Dv: 0.384	St.Dv: 0
*13*	Stand on heels	Full score: 77%	Full score: 90%	Full score: 91%	Full score: 92%
		Mean: 1.742	Mean: 1.901	Mean: 1.909	Mean: 1.923
		St.Dv: 0.505	St.Dv: 0.300	St.Dv: 0.291	St.Dv: 0.269
*11*	Rise from floor	Full score: 40%	Full score 65%	Full score 97%	Full score: 92%
		Mean: 1.400	Mean: 1.647	Mean: 1.969	Mean: 1.923
		St.Dv: 0.497	St.Dv: 0.483	St.Dv: 0.174	St.Dv: 0.269
*12*	Lifts head	Full score: 43%	Full score: 75%	Full score: 88%	Full score: 92%
		Mean: 1.382	Mean: 1.745	Mean: 1.878	Mean: 1.923
		St.Dv: 0.603	St.Dv: 0.440	St.Dv: 0.331	St.Dv: 0.269
*15*	Hop—R	Full score: 23%	Full score: 67%	Full score: 88%	Full score: 88%
		Mean: 1.057	Mean: 1.588	Mean: 1.878	Mean: 1.884
		St.Dv: 0.639	St.Dv: 0.638	St.Dv: 0.331	St.Dv: 0.322
*16*	Hop—L	Full score: 14%	Full score: 69%	Full score: 88%	Full score: 90%
		Mean: 0.857	Mean: 1.627	Mean: 1.878	Mean: 1.903
		St.Dv: 0.648	St.Dv: 0.598	St.Dv: 0.331	St.Dv: 0.297
*TOT*		Mean: 28.371	Mean: 31.843	Mean: 33.303	Mean: 33.519
		St.Dv: 3.581	St.Dv: 2.318	St.Dv: 1.015	St.Dv: 0.851
		Max: 34	Max: 34	Max: 34	Max: 34
		Min: 20	Min: 25	Min: 31	Min: 31

The comparison with available developmental scales for which age related reference data (were available confirmed that the activities in the individual items (12–13) were compatible with what observed in our study on controls.

On the basis of these results, the scale was designed rearranging the order of the items. The first part of the revised scale only includes the 8 items that can be administered at 3 years, the second part includes the additional 5 items that can be assessed at 3.5 years and the final part the last 4 items that can be assessed after the age of 4 years (Table 2).

**Table 2:** Revised version of the NSAA with items ordered according to the age when they can be performed.

	ACTIVITY	2	1	0
1	**Stand**	Stands upright, still and symmetrically, without compensation (with heels flat and legs in neutral) for minimum count of 3 secs	Stands still but with some degree of compensation (e.g. on toes or with legs abducted or with bottom stuck out) for minimum count of 3 secs	Cannot stand still or independently, needs support (even minimal)
2	**Walk**	Walks with heel-toe or flat-footed gait pattern	Persistent or habitual toe walker, unable to heel-toe consistently	Loss of independent ambulation—may use KAFOs or walk short distances with assistance
3	**Stand up from chair**	Keeping arms folded. Starting position 90o hips and knees, feet on floor/supported on a box step.	With help from thighs or push on chair or prone turn	Unable
6	**Climb box step- Right**	Faces step—no support needed	Goes up sideways or needs support	Unable
7	**Climb box step- Left**	Faces step—no support needed	Goes up sideways or needs support	Unable
10	**Gets to sitting**	Starts in supine—may use one hand to assist	Self assistance e.g.–pulls on legs or uses head-on- hands or head flexed to floor	Unable
14	**Jump**	Both feet at the same time, clear the ground simultaneously	One foot after the other (skip)	Unable
17	**Run**	Both feet off the ground (no double stance phase during running)	‘Duchenne jog’	Unable
			**TOTAL 3 years (max score 16)**	
4	**Stand on one leg—Right**	Able to stand in a relaxed manner (no fixation) for count of 3 seconds	Stands but either momentarily or needs a lot of fixation e.g. by knees tightly adducted or other trick	Unable
5	**Stand on one leg—Left**	Able to stand in a relaxed manner (no fixation) for count of 3 seconds	Stands but either momentarily or needs a lot of fixation e.g. by knees tightly adducted or other trick	Unable
8	**Descend box—Right**	Faces forward, climbs down controlling weight bearing leg. No support needed	Sideways, skips down or needs support	Unable
9	**Descend box—Left**	Faces forward, climbs down controlling weight bearing leg. No support needed	Sideways, skips down or needs support	Unable
13	**Stands on heels**	Both feet at the same time, clearly standing on heels only (acceptable to move a few steps to keep balance) for count of 3	Flexes hip and only raises forefoot	Unable
			**TOTAL 3.5years (max score 26)**	
11	**Rise from floor**	From supine—no evidence of Gowers’ manoeuvre*	Gowers’ evident	(a) NEEDS to use external support object e.g. chair OR (b) Unable
12	**Lifts head**	In supine, head must be lifted in mid-line. Chin moves towards chest	Head is lifted but through side flexion or with no neck flexion	
15	**Hop—Right**	Clears forefoot and heel off floor	Clears forefoot and heel off floor	Unable
16	**Hop—Left**	Clears forefoot and heel off floor	Clears forefoot and heel off floor	Unable
			**TOTAL 4 years and above (max score 34)**	

### DMD cohort

23 patients had an assessment at 3 years, their total scores ranged between 8 and 16 (mean total score: 11, SD: 2.16). 44 patients had an assessment at 3.5 years, their total scores ranged between 4 and 25 (mean total score: 16, SD: 3.84); 46 patients had an assessment at 4 years, their total scores ranged between 6 and 33 (mean total score: 20, SD: 5.96); 60 patients had an assessment at 4.5 years, their total scores ranged between 10 and 34 (mean total score: 22, SD: 6.09). Details of the scores in the whole cohort are provided in S1 Table. Their mean % predicted scores went from 69% at 3 years, to 61, 59 and 65% at the subsequent ages.

**Fig 1:**
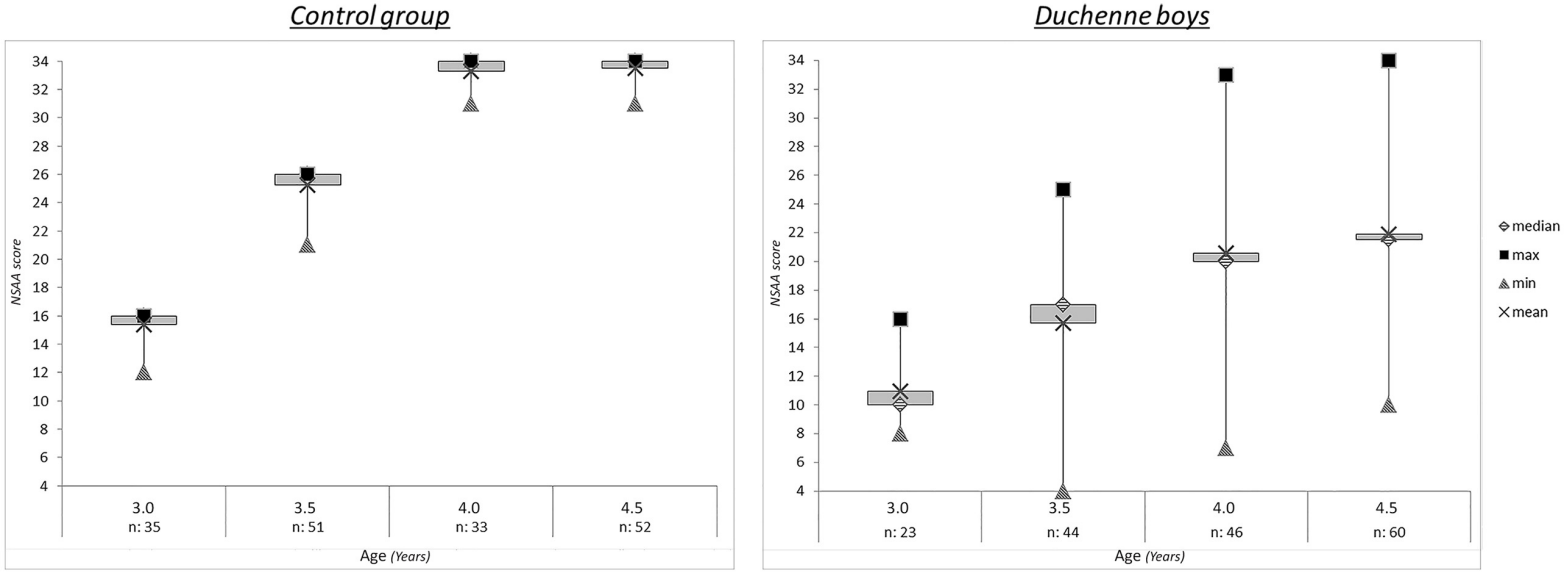
NSAA scores in controls boys compare to DMD NSAA scores subdivided by age of evaluation.

Fig 1 shows median, and ranges of the scores in DMD compared to the controls.

### Comparison between typically developing and DMD boys

When we compared the individual items and the total score in each age subgroup in typically developing versus DMD boys (Tables 1 and 2), we found that DMD boys showed significantly lower scores compared to their age matched controls in the total score and in the individual items, with the exception of items 1, 2, 3, 9 and 12 in the 3 year group and items 1, and 2 in the other subgroups.

## Discussion

Over the last few years there has been increasing concern about the possibility to find reliable outcome measures in young DMD. The few published studies have demonstrated that motor activities and, more generally motor development, can be measured, but a number of issues should be taken into account. Developmental scales provide the opportunity to follow longitudinally young boys and to obtain developmental quotients using age appropriate reference data at each age point [15–16]. The problem with these scales is that they include a wide range of items that are not always relevant to DMD and can only be used up to a certain age. In contrast, functional motor scales, like the NSAA are not appropriate for children below the age of 4 and have only been validated for boys above the age of 5. Other scales, like the MFM have also been validated from the age of 5 years [17].

In order to fill this gap, we adapted the NSAA to make it suitable for boys younger than 5 years. We first assessed all the NSAA 17 items in a cohort of typically developing children of age between 3 years and 4.5 years (+3 months), subdivided according to their age age at 6 month intervals. At each age point, we included only the items that were age appropriate, as they could be completed by more than 85% of the controls, excluding the others. This resulted in a scale including only 8 items for boys at 3 years, 13 items at 3.5 while, by the age of 4 all the original items could be included. The appropriateness of the items selected at each age point was confirmed looking at the age reference of the same activities on different neurodevelopmental assessments.

The revised version combines the advantages of the neurodevelopmental scales, allowing to assess the level of function in relation to age, with the properties of the original NSAA scale. Unlike neurodevelopmental scales, that include a range of activities that are not specific to DMD, the NSAA scale items were specifically selected to assess activities reflecting the pattern of muscle weakness found in DMD. Furthermore, the scoring system allows to score not only if the boys are able to perform a task (score 2) but also if the task can be achieved using other strategies (score 1), that is very common in DMD and allows better chances to observe possible changes following intervention.

Another advantage of the revised version compared to neurodevelopmental scales is that while those can be used only in the first years, the NSAA, in its revised version, can be used from the age of 3 years until they lose ambulation.

The application of the revised version of the scale to our retrospective DMD data confirmed that young DMD boys often acquire the ability to perform new abilities with increasing age but only few of the DMD boys, at any age, achieve a full total score for their age. Our cross sectional study show how the raw scores were progressively higher in boys from the age of 3 to 4.5 years. At 4 years the scores were higher than at 3 and 3.5 but the score adjusted for age, using % predicted, were lower with increasing age as they do not achieve the skills gained by typically developing boys of the same age. After the age of 4, both the raw and % predicted scores appeared to be higher than that found in younger boys. Although these findings should be interpreted with caution as this was not a longitudinal study, they appear to confirm longitudinal data obtained using the Bayley scales in boys between the age of 2 and 3 years suggesting that in the early phases of development DMD boys acquire many activities but with a delay [5]. Interestingly, the activities that showed improvement, suggesting a possible delay, were climbing or descending steps while others, such as hopping or running fast, could not be achieved even at the age of 4 or 4.5 years. This is not surprising as in DMD these are generally only achieved in boys on steroids and these were not included in this paper.

In conclusion, our study suggests that a revised version of the NSAA can be used in boys from the age of 3 years to assess early functional changes and obtain information on how young DMD boys acquire new abilities with increasing age and how this correlates with their peers. Further studies, using longitudinal data will help to better understand the range of changes and to identify possible changes related to type and site of mutations or to the use of steroids or other interventions that are increasingly used in boys younger than 4 years. Further studies, collecting larger datasets, will also help to establish if the revised scale maintains the psychometric properties of the full NSAA demonstrated by Rasch analysis.

## Supporting Information

S1 Table DMD boys on individual NSAA items at different age points. The shaded cells indicate the activities that were achieved by the boys referring to the revised scale items at that age point.
(PDF)
