# Supplementary material for: Revised North Star Ambulatory Assessment for Young Boys with Duchenne Muscular Dystrophy
Source: PLoS One. 2016 Aug 5;11(8):e0160195. doi: 10.1371/journal.pone.0160195 (PMC4975396; doi:10.1371/journal.pone.0160195)
Supplement: S1 Table — The shaded cells indicate the activities that were achieved by the boys referring to the revised scale items at that age point. (PDF) [file pone.0160195.s001.pdf]

	North Star	DMD<br>3 years (n:23)	DMD<br>3.5 years(n:44)	DMD<br>4 years(n:46)	DMD<br>4.5 years(n:60)
1	Stand	Full score 100%<br>Mean 2<br>St.Dv 0	.Full score 98%<br>Mean 1.977<br>St.Dv 0.150	Full score 93%<br>Mean 1.934<br>St.Dv 0.249	Full score 95%<br>Mean 1.950<br>St.Dv 0.219
2	Walk (10m)	Full score 96%<br>Mean 1.956<br>St.Dv 0.208	Full score 95%<br>Mean 1.954<br>St.Dv 0.210	Full score 93%<br>Mean 1.934<br>St.Dv 0.249	Full score 97%<br>Mean 1.966<br>St.Dv 0.181
3	Sit to stand from chair	Full score 78%<br>Mean 1.782<br>St.Dv 0.421	Full score 80%<br>Mean 1.795<br>St.Dv 0.408	Full score 83%<br>Mean 1.826<br>St.Dv 0.383	Full score 85%<br>Mean 1.833<br>St.Dv 0.418
6	Climb step - R	Full score 35%<br>Mean 1.304<br>St.Dv 0.558	Full score 27%<br>Mean 1.204<br>St.Dv 0.553	Full score 63%<br>Mean 1.586<br>St.Dv 0.580	Full score 55%<br>Mean 1.533<br>St.Dv 0.535
7	Climb step – L	Full score 26%<br>Mean 1.173<br>St.Dv 0.576	Full score 25%<br>Mean 1.181<br>St.Dv 0.540	Full score 59%<br>Mean 1.543<br>St.Dv 0.585	Full score 57%<br>Mean 1.550<br>St.Dv 0.534
10	Gets to sitting	Full score 52%<br>Mean 1.521<br>St.Dv 0.510	Full score 52%<br>Mean 1.454<br>St.Dv 0.627	Full score 43%<br>Mean 1.391<br>St.Dv 0.576	Full score 55%<br>Mean 1.559<br>St.Dv 0.500
14	Jump	Full score 13%<br>Mean 0.454<br>St.Dv 0.738	Full score 23%<br>Mean 0.720<br>St.Dv 0.825	Full score 48%<br>Mean 1.133<br>St.Dv 0.919	Full score 48%<br>Mean 1.241<br>St.Dv 0.844
17	Run	Full score 17%<br>Mean 0.782<br>St.Dv 0.735	Full score 27%<br>Mean 0.930<br>St.Dv 0.798	Full score 28%<br>Mean 1.044<br>St.Dv 0.737	Full score 30%<br>Mean 1.133<br>St.Dv 0.675
4	Stand on one leg - R	Full score 9%<br>Mean 0.600<br>St.Dv 0.680	Full score 16%<br>Mean 0.853<br>St.Dv 0.691	Full score 22%<br>Mean 1.022<br>St.Dv 0.664	Full score 40%<br>Mean 1.275<br>St.Dv 0.695
5	Stand on one leg – L	Full score 13%<br>Mean 0.700<br>St.Dv 0.732	Full score 14%<br>Mean 0.829<br>St.Dv 0.667	Full score 26%<br>Mean 1.068<br>St.Dv 0.695	Full score 35%<br>Mean 1.206<br>St.Dv 0.694
8	Descend step - R	Full score 22%<br>Mean 1.130<br>St.Dv 0.548	Full score 25%<br>Mean 1.227<br>St.Dv 0.475	Full score 57%<br>Mean 1.521<br>St.Dv 0.586	Full score 52%<br>Mean 1.466<br>St.Dv 0.595
9	Descend step – L	Full score 26%<br>Mean 1.173<br>St.Dv 0.576	Full score 16%<br>Mean 1.0681818<br>St.Dv 0.501	Full score 46%<br>Mean 1.413<br>St.Dv 0.580	Full score 52%<br>Mean 1.483<br>St.Dv 0.567
13	Stand on heels	Full score 9%<br>Mean 0.421<br>St.Dv 0.692	Full score 11%<br>Mean 0.4615385<br>St.Dv 0.719	Full score 28%<br>Mean 0.904<br>St.Dv 0.849	Full score 30%<br>Mean 0.964<br>St.Dv 0.830
11	Rise from floor	Full score 0%<br>Mean 1<br>St.Dv 0	Full score 11%<br>Mean 1.068<br>St.Dv 0.397	Full score 7%<br>Mean 1.051<br>St.Dv 0.320	Full score 15%<br>Mean 1.133<br>St.Dv 0.389
12	Lifts head	Full score 22%<br>Mean 0.956<br>St.Dv 0.705	Full score 41%<br>Mean 1.159<br>St.Dv 0.805	Full score 37%<br>Mean 1.119<br>St.Dv 0.832	Full score 33%<br>Mean 1.133<br>St.Dv 0.724
15	Hop – R	Full score 0%<br>Mean 0<br>St.Dv 0	Full score 2%<br>Mean 0.09<br>St.Dv 0.374	Full score 4%<br>Mean 0.175<br>St.Dv 0.500	Full score 8%<br>Mean 0.379<br>St.Dv 0.644
16	Hop - L	Full score 0%<br>Mean 0<br>St.Dv 0	Full score 2%<br>Mean 0.121<br>St.Dv 0.399	Full score 7%<br>Mean 0.200<br>St.Dv 0.563	Full score 8%<br>Mean 0.344<br>St.Dv 0.636
TOT		Mean 10.960<br>St.Dv 2.160<br>Max 16<br>Min 8	Mean 15.710<br>St.Dv 3.840<br>Max 25<br>Min 4	Mean 20.560<br>St.Dv 5.960<br>Max 33<br>Min 6	Mean 21.900<br>St.Dv 6.090<br>Max 34<br>Min 10
